# The complete mitochondrial genome and phylogenetic analysis of *Ischnochiton hakodaensis* (Carpenter, 1893)

**DOI:** 10.1080/23802359.2019.1642164

**Published:** 2019-07-17

**Authors:** Yutong Cui, Xiaoyu Guo, Shanshan Wang, Yanran Xu, Xiaoyue Sun, Ruoran Li, Yunhui Wang, Jiangyong Qu, Xumin Wang, Xiumei Liu

**Affiliations:** College of Life Sciences, Yantai University, Yantai, China

**Keywords:** *Ischnochiton hakodaensis*, Polyplacophora, phylogenetic analysis, mitochondrial genome

## Abstract

*Ischnochiton hakodaensis* is one of Polyplacophora species, which plays an important role in the intertidal and subtidal ecosystems. In this study, the complete mitochondrial genome of *I. hakodaensis* was obtained with 15,139 bp in length, including 13 protein-coding genes (PCGs), 22 transfer RNA (tRNA) genes, 2 ribosomal RNA (rRNA) genes. The overall base composition of the genome is 35.93% A, 13.51% G, 37.19% T, 13.38% C. The phylogenetic tree show that *I. hakodaensis*, *Acanthopleura brevispinosa*, *Acanthopleura granulate*, and *Liolophura japonica* constituted a sister clade along with *Tonicia forbesii* and *Tonicia lamellose*.

## Introduction

Chitons are marine molluscs with eight articulated shell plates encircled by a muscular girdle (Palmer 2012). *Ischnochiton hakodaensis* is an important species of chitons, which plays a vital role in the intertidal and subtidal ecosystems (Dou et al. 2018). In this study, we obtained the complete mitochondrial genome of *I. hakodaensis*. The specimen was collected from Beihuangcheng Island, Shandong Province, China (38°23′2″N, 120°55′34″E), and immediately immersed in 100% ethanol, and then stored in a refrigerator at −80 °C (Pu et al. 2017). The specimen was deposited in the marine specimen room of Yantai University with an accession number YTU-SKY-20181008. Based on the manufacturer’s protocol, the genomic DNA was extracted from the muscle tissue by DNeasy Blood & Tissue Kit (Qiagen, Hilden, Germany). The llumina Hiseq 4000 sequencing system (Illumina, San Diego, CA) was used to analyze the mitochondrial genome sequences of *I*. *hakodaensis*. The mitogenome of *I*. *hakodaensis* was annotated by the mitochondrial genome annotation server (Bernt et al. 2013) and tRNAscan-SE server (Lowe and Chan 2016).

The complete mitogenome of *I. hakodaensis* was composed as a circular molecule of 15,139 bp (GenBank accession number KY827038) with a nucleotide composition of 35.93% A, 13.51% G, 37.19% T, 13.38% C, and contained an overall A + T content of 73.12%. The mitogenome of *I. hakodaensis* contained 13 protein-coding genes (PCGs), 22 transfer RNA (tRNA) genes, and 2 ribosomal RNA (rRNA) genes. All genes show the typical gene arrangement conforming to the Mollusca consensus (Noack et al. 1996). The seven PCGs (*COX1*, *COX2*, *COX3, ATP8*, *Cytb*, *NAD4L*, and *NAD1*) use the typical ATG as the start codon, the rest seven genes use ATA as the initiation codon. Two genes (*NAD4L* and *NAD1*) use TAG as the stop codon, and the other 11 genes end with TAA. All tRNAs have the typical cloverleaf structure ranged from 64–71 bp.

**Figure 1.**
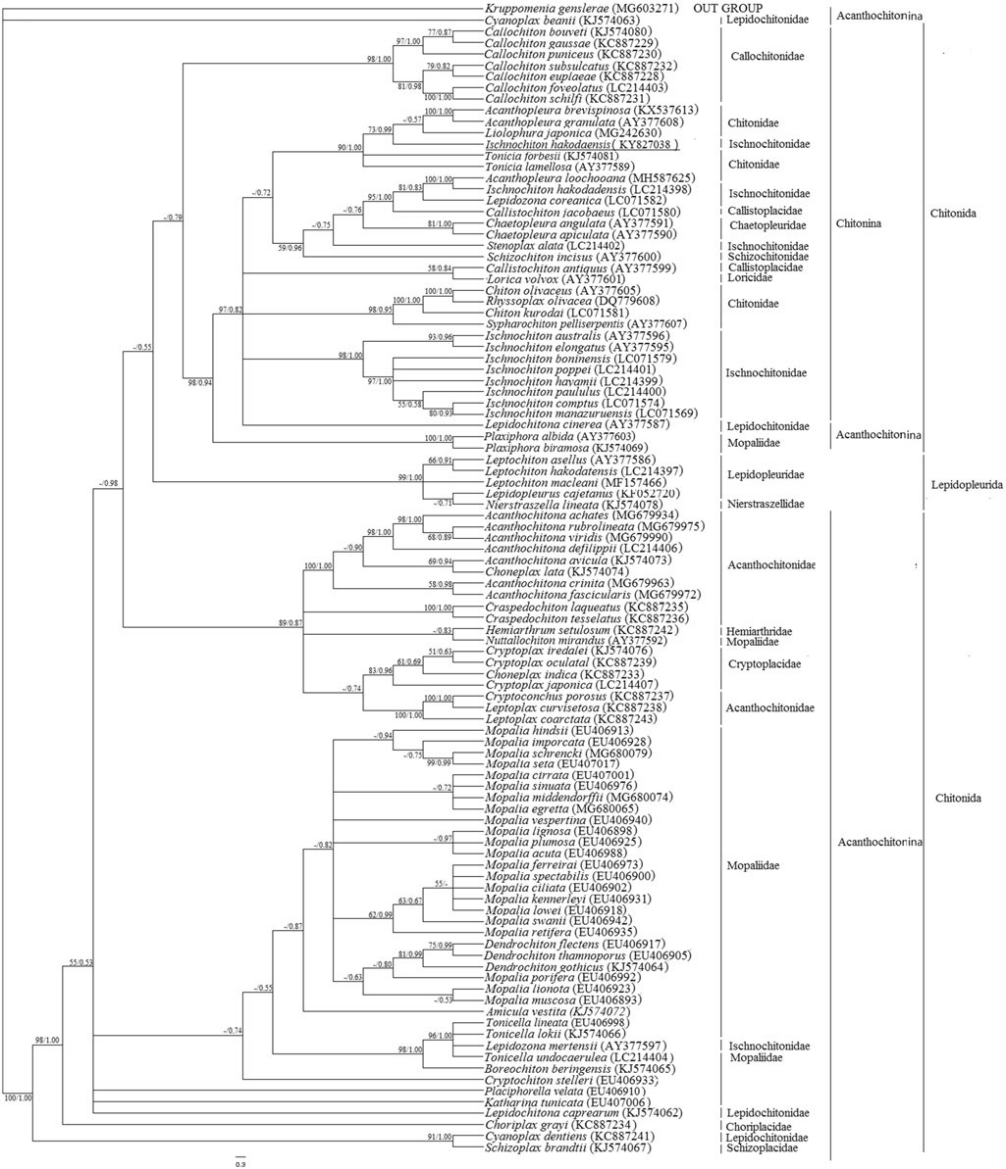
Bayesian tree was constructed by 16S ribosomal RNA (16S rRNA) of 101 Polyplacophora species. The bootstrap values for the BI and ML analysis were shown on the nodes (left is ML bootstrap values, right is BI bootstrap values, ‘-’ means the bootstrap values less than 50%). The underlined markers represent the species *Ischnochiton hakodaensis* in this study. The brackets after the species mean Accession number from GenBank.

The phylogenetic tree was constructed by 16S ribosomal RNA (16S rRNA) of 101 Polyplacophora species using Bayesian inference (BI) (Huelsenbeck and Ronquist 2001) and maximum likelihood (ML) (Guindon et al. 2009) methods. The BI and ML trees show similar topological structure (Figure 1). *Ischnochiton hakodaensis* was phylogenetically distinct from the other eight *Ischnochiton* species constituting one monophyletic group. *Ischnochiton hakodaensis*, *Acanthopleura brevispinosa*, *Acanthopleura granulate*, and *Liolophura japonica* constituted a sister clade along with *Tonicia forbesii* and *Tonicia lamellose* (Figure 1). The taxonomic status of Polyplacophora species should be further evaluated.
